# Longitudinal and prospective assessment of prenatal maternal sleep quality and associations with newborn hippocampal and amygdala volume

**DOI:** 10.1016/j.dcn.2022.101174

**Published:** 2022-11-09

**Authors:** Melissa Nevarez-Brewster, Catherine H. Demers, Alexandra Mejia, Mercedes Hoeflich Haase, Maria M. Bagonis, Sun Hyung Kim, John H. Gilmore, M. Camille Hoffman, Martin A. Styner, Benjamin L. Hankin, Elysia Poggi Davis

**Affiliations:** a University of Denver, Department of Psychology, United States; b University of Colorado Anschutz Medical Campus, Department of Psychiatry, United States; c University of North Carolina – Chapel Hill, Department of Psychiatry, United States; d University of North Carolina – Chapel Hill, Department of Computer Science, United States; e University of Colorado Denver School of Medicine, Department of Obstetrics and Gynecology, Division of Maternal and Fetal Medicine, United States; f University of Illinois at Urbana-Champaign, Department of Psychology, United States; g University of California, Irvine, Department of Pediatrics, United States

**Keywords:** Pregnancy, Sleep, Newborn neurodevelopment, Amygdala, Hippocampus, MRI

## Abstract

**Background:**

The rapid maturation of the fetal brain renders the fetus susceptible to prenatal environmental signals. Prenatal maternal sleep quality is known to have important health implications for newborns including risk for preterm birth, however, the effect on the fetal brain is poorly understood.

**Method:**

Participants included 94 pregnant participants and their newborns (53% female). Pregnant participants (*M*_age_ = 30; *SD*_age_*=* 5.29) reported on sleep quality three times throughout pregnancy. Newborn hippocampal and amygdala volumes were assessed using structural magnetic resonance imaging. Multilevel modeling was used to test the associations between trajectories of prenatal maternal sleep quality and newborn hippocampal and amygdala volume.

**Results:**

The overall trajectory of prenatal maternal sleep quality was associated with hippocampal volume (left: *b* = 0.00003, *p* = 0.013; right: *b* = 0.00003, *p* = .008). Follow up analyses assessing timing of exposure indicate that poor sleep quality early in pregnancy was associated with larger hippocampal volume bilaterally (e.g., late gestation left: *b* = 0.002, *p* = 0.24; right: *b* = 0.004, *p* = .11). Prenatal sleep quality was not associated with amygdala volume.

**Conclusion:**

These findings highlight the implications of poor prenatal maternal sleep quality and its role in contributing to newborn hippocampal development.

## 1 Introduction

The prenatal period is characterized by exceptionally rapid maturation. In the span of nine months, the single-celled zygote becomes a human newborn capable of regulating and sustaining homeostatic processes (Demers et al., 2021a, Thomason, 2020). The fetal brain transforms rapidly, with the fetus forming approximately 200 billion neurons by the end of the second trimester (Ackerman, 1992). Neurogenesis commences around nine weeks into gestation (Bourgeois, 1997, Kolb and Fantie, 2008); neurons are produced at a rate of more than 300,000 nerve cells per minute (Ackerman, 1992). Such rapid intrauterine development increases fetal susceptibility to prenatal environmental signals. As proposed by the Fetal or Developmental Origins of Adult Disease Hypothesis, environmental signals can promote or jeopardize fetal development, altering the maturation of physiological systems with lifelong consequences for health and disease (Barker, 1998, Barker, 2002, Kwon and Kim, 2017).

Poor sleep quality and short sleep duration are public health concerns that may have intergenerational consequences. During pregnancy, approximately 75% of people experience worsening sleep quality (Lucena et al., 2018, Mindell et al., 2015), making poor prenatal sleep health a pervasive problem. Disturbed sleep during pregnancy can impact offspring physical health including preterm birth and low birth weight as well as high blood pressure and body mass index in childhood (Okun et al., 2011, Wang et al., 2017, Harskamp-van Ginkel et al., 2020). Only a handful of studies have looked at the associations between prenatal maternal sleep and postnatal outcomes in humans. Prenatal maternal sleep predicts newborn event related potential (ERP) responses to auditory stimuli (Lavonius et al., 2020), infant negative affectivity (Ciciolla et al., 2022), and socioemotional development (Trauman et al., 2015). Recent reviews have identified prenatal maternal sleep health as an understudied and potentially critical process that may influence the developing fetus (Johnson and Louis, 2022, Mindell et al., 2015, Moreno-Fernandez et al., 2020). While the pathways by which sleep disturbances impact the fetus are unknown, maternal sleep disruptions impact physiological processes such as inflammation and stress responsivity (Bleker et al., 2017, Bublitz et al., 2018, Okun et al., 2007), which are known to shape fetal brain development (Davis et al., 2020b; Davis et al., 2017; Graham et al., 2018; Sandman et al., 2018).

Growing preclinical literature indicates that poor prenatal maternal sleep impacts offspring neurodevelopment in rodents (Vanderplow et al., 2022) and that the developing hippocampus, a region involved in memory consolidation and learning (Han et al., 2020, Peng et al., 2016), may be particularly susceptible (Argeri et al., 2016, Pires et al., 2020). Sleep deprivation in pregnant dams causes impairments in hippocampus-dependent memory in the offspring (Pires et al., 2021). Evaluation of underlying mechanisms illustrates that maternal sleep deprivation compromises offspring hippocampal function (Zhao et al., 2015), including neurogenesis during both prepubescence (Han et al., 2020, Zhao et al., 2014) and adulthood (Motta-Teixeira et al., 2018). Effects can be detected as early as infancy as pups born to sleep-deprived dams display compromised hippocampal neurogenesis (Peng et al., 2016) and synaptic plasticity (Yu et al., 2018) compared to controls. Together, these findings demonstrate that prenatal maternal sleep may sculpt neurogenesis and synaptic plasticity, thus altering neural circuit development.

Guided by preclinical work indicating that sleep disruption affects the hippocampus, the present study examined the impact of prenatal maternal sleep health on newborn hippocampal volume. The hippocampus largely develops *in utero*, with major cell proliferation commencing around the fourth week of gestation and progressing to the development of hippocampal fissures by the 22nd week of gestation (Thomason, 2020) and hippocampal development is susceptible to prenatal environmental influences (Avishai-Eliner et al., 2002, Bock et al., 2015). Although the link between prenatal maternal sleep and fetal hippocampal development is unknown in humans, research with non-pregnant individuals suggests that hippocampal development is susceptible to sleep disruptions (Akers et al., 2018, Mirescu et al., 2006). For these reasons, the hippocampus was identified as the primary region of interest.

Similar to the hippocampus, the amygdala is another region that is susceptible to prenatal perturbations (e.g., prenatal stress and inflammation; Buss et al., 2012; Graham et al., 2018). The amygdala, implicated in fear and emotion regulation (Barrett et al., 2007, Feng et al., 2018), undergoes substantial development *in utero* and is among the first of the subcortical regions to develop embryonically, with its earliest traces found around seven gestational weeks (Buss et al., 2012, Humphrey, 1968). Unlike the hippocampus, prenatal sleep has not been linked to offspring amygdala volume. However, because the prenatal environment has been linked to development of the amygdala (Rifkin-Graboi et al., 2013, Demers et al., 2022) and as evidence suggests poor sleep health is associated with amygdala function in non-pregnant populations (Prather et al., 2013, Yoo et al., 2007), the amygdala was included as a secondary outcome of interest.

The present study investigates the link between prenatal maternal sleep quality and newborn hippocampal and amygdala volume. Assessing hippocampal and amygdala morphology shortly after birth allows for the identification of prenatal influences prior to the intervening effects of postnatal life (Demers et al., 2022, Demers et al., 2021b). Further, sleep quality is dynamic over pregnancy and there are dramatic changes in fetal brain development across gestation (Lyu et al., 2020; Nevarez-Brewster, 2022; Whitaker et al., 2021). Sleep disruptions early in gestation are strongly linked to preterm birth (Okun et al., 2011), and rodent work suggests that the timing of exposure to prenatal maternal sleep disturbances differentially affects offspring behavioral outcomes in pups exposed to sleep deprivation early and late gestation (Peng et al., 2016). Thus, there may be sensitive windows for timing of exposure to prenatal sleep deprivation. We, therefore, evaluated whether links between prenatal maternal sleep quality and newborn hippocampal and amygdala volume differ based on timing of exposure.

## 2 Materials and methods

### 2.1 Study Overview

Pregnant participants reported on sleep quality longitudinally throughout gestation and provided demographic information. Subjective maternal prenatal sleep quality was assessed three times between eight and 39 gestational weeks (*M*_*Gestational Age (GA) early*_= 16.9, *SD*_*GA early*_= 4.3), (*M*_*GA middle*_= 28.4, *SD*_*GA middle*_= 3.8), (*M*_*GA late*_= 35.3, *SD*_*GA late*_= 1.6). Neonatal hippocampal and amygdala volume were assessed during natural sleep at 44 postconceptional weeks (*M*= 44.22, *SD*= 2.56, range = 42–56 weeks). Postconceptional weeks was defined as the sum of weeks' gestation at birth and weeks from birth to the MRI scan. Participants were compensated at each time point in which data was collected. This study was approved by the University of Denver and the Colorado Multiple Institutional Review Board, and all participants provided written and informed consent.

### 2.2 Participants

Participants included 94 pregnant individuals and their newborns from the Care Project, a longitudinal study investigating the influence of maternal mental health during pregnancy on offspring developmental outcomes (see Davis et al., 2018 for more details). Participants were recruited from obstetrics and gynecology clinics in and around Denver, Colorado. Participants meeting inclusion criteria were contacted by a research assistant, the study protocol was described, and interested participants were then consented. Initial inclusion criteria for participants’ enrollment in the study were a) maternal age between 18 and 45 years, b) singleton pregnancy, c) gestational age (GA) less than 25 weeks at time of enrollment, and d) proficiency in English. Initial exclusion criteria at recruitment included a) current drug or methadone use, b) major health conditions requiring invasive treatments (e.g., dialysis, blood transfusions, chemotherapy), c) current or past symptoms of psychosis or mania based on the Structured Clinical Interview (SCID) for DSM-5, and d) current participation in cognitive behavioral therapy or interpersonal therapy. Additional exclusion criteria for the current study included a) miscarriage or fetal demise of the current pregnancy (*n* = 2), b) major fetal or chromosomal anomalies (*n* = 1) or neonatal complications requiring a NICU stay (*n* = 0) and c) any MRI contraindications (*n* = 4; e.g., oxygen support). Of the 101 newborns who attended the MRI scan, six were unable to be scanned (e.g., the newborn did not fall asleep during the scanning window), and one did not yield imaging data (e.g., the newborn woke up in the scanner). Thus, 94 mother-newborn dyads were included in the present study. The newborns that were unable to be scanned did not differ from the remaining sample on income, gestational age at birth, and cohabitation status (all *t*s < 2.02, all *p*s > .09).

Pregnant participants were, on average, 30 years old (*SD*_age_*=* 5.29) at time of enrollment. More than half (56.4%) obtained at least a college degree and the majority (89.4%) were cohabitating with a partner at time of enrollment. Participants identified as 58% non-Latinx White, 16% Latinx/Hispanic, 11% African American/Black, 3% Asian American/Asian, 1% Native Hawaiian/Pacific Islander, and 11% multiracial.^2^ Further, participants reported a median household annual income of $72,000, with 29% of participants living at or below the 200% federal poverty line. Five percent (*n* = 4) of pregnant participants reported prenatal substance use. Newborns (53.2% female) were, on average, 39 gestational weeks at birth (range= 34.86–41.71). See Table 1 for more participant details.

**Table 1:** Sample Characteristics (N = 94).

Maternal characteristics	*M (SD) or %*
Age at enrollment	30.45 (5.29)
Obstetric complications	
No complications	33.3%
One complication	38.7%
Two or more complications	28%
Annual household income ($)	72,000 (50,117.26)^a^,^b^
Household INR	3.56 (2.89)^a^,^b^
Cohabitation status	
Cohabitating with partner	89.4%
Living alone	9.6%
Other	1.1%
Education (highest degree earned)	
Less than high school	2.2%
High school	10.6%
Some college	18.1%
Associate degree	12.8%
Bachelor's degree	34%
Graduate degree	22.3%
Race and ethnicity	
Asian American/Asian	3%
African American/Black	11%
Hispanic/Latinx	16%
Non-Latinx White	58%
Native Hawaiian/Pacific Islander	1%
Multiracial/Multiethnic	11%
Prenatal Substance Use	
Marijuana	2.1%
Alcohol	2.1%
Cigarettes	1.1%
**Newborn Characteristics**	
Postconceptional age at MRI (weeks)	44.22 (2.56)
Biological sex at birth (% female)	53.2%
Race and ethnicity	
Asian American/Asian	3%
African American/Black	8%
Hispanic/Latinx	20%
Non-Latinx White	46%
Native Hawaiian/Pacific Islander	1%
Multiracial/Multiethnic	22%
**Birth outcome**	
Gestational age at birth (weeks)	39.12 (1.29)
Birth weight percentile	46.01 (25.44)
5-minute Apgar score	8.81 (0.47)
**Study variables**	
PSQI at 1st timepoint	6.40 (3.70)
PSQI at 2nd timepoint	6.77 (3.68)
PSQI at 3rd timepoint	7.63 (3.84)
Right hippocampus (mm^3^)	1154.17 (153.90)
Left hippocampus (mm^3^)	1106.10 (152.58)
Right amygdala (mm^3^)	246 (32.21)
Left amygdala (mm^3^)	239.70 (33.04)
Intracranial volume (mm^3^)	544369.33 (61510.09)

Note:

a median used,

b An outlier for income (i.e., SD ≥ 5 above the mean) was converted to the value 3 SDs above the mean, preserving its rank as the highest value; INR = Income to needs ratio, MRI = magnetic resonance imaging, NICU = neonatal intensive care unit

### 2.3 Measures

#### 2.3.1 Sleep quality

Prenatal maternal sleep quality was collected using the Pittsburg Sleep Quality Index (PSQI; Buysse et al., 1989). The PSQI is a 19-item self-report questionnaire consisting of seven subscales (sleep latency, sleep duration, sleep disturbances, sleep medication, subjective sleep quality, sleep efficiency, and daytime dysfunction), weighed on a 0–3 scale. The subscale scores are then added, yielding an overall subjective sleep quality score that ranges from zero to 21. Higher scores are indicative of poor sleep quality (Buysse et al., 1989). The PSQI has previously demonstrated acceptable internal reliability (*α* = .83; Buysse et al., 1989). Additionally, the PSQI possesses good convergent and discriminant validity when used early in pregnancy (Zhong et al., 2015, Skouteris et al., 2009, Jomeen and Martin, 2007). In our sample, 63% percent of participants reflected “poor sleeper” scores (PSQI score ≥ 5; Tomfohr et al., 2015) at the beginning of pregnancy. The percentage of “poor sleepers” increased from 63% in the beginning of pregnancy to 81% late in pregnancy, which is consistent with existing findings (Lucena et al., 2018). Internal consistency of PSQI subscales was acceptable across all timepoints (all *α*s > .73). In our sample, one participant had missing sleep data in early (1%), and in middle (1%) pregnancy, and seven later in pregnancy (7%). Gestational age at each prenatal assessment was computed using the date of PSQI completion and used to compute PSQI trajectories based on gestational weeks at assessment.

#### 2.3.2 Magnetic resonance imaging acquisition

Newborns were scanned during natural and unsedated sleep. A Siemens Skyra 3 T MRI system equipped with a 20-channel head coil at the Brain Imaging Center at the University of Colorado Anschutz Medical Campus was used. Prior to scanning, newborns were fed, swaddled, and placed into the scanner with their heads secured in a vacuum-fixation device to limit scan noise due to motion. Newborns wore earplugs and headphones to prevent wakefulness from the acoustic noise of the scan. Newborns were monitored by a research staff member who was in the scanner for the entirety of the scan and caregivers remained in the scan room if they chose to do so.

T1-weighted (T1w) images were obtained using a three-dimensional magnetization-prepared rapid gradient echo sequence (repetition time = 1900 ms; echo time = 3.07 ms; inversion time = 900 ms; flip angle 9°; 4 min 26 s) and T2-weighted (T2w) images were obtained with a 3D fast turbo spin echo sequence (repetition time 3200 ms; echo time = 408 ms; flip angle var; 4 min 43 s). The spatial resolution was a 0.82 × 0.82 × 0.8 mm voxel for T1w and 0.86 mm × 0.86 mm × 0.8 mm voxel for T2w.

#### 2.3.3 Magnetic resonance imaging processing

Image quality control (QC) feedback was provided using a four-point scale (0−3) (Blumenthal et al., 2002) adapted in-house for newborn scanning (Gilmore et al., 2020). Criteria for exclusion was a QC score of 0, indicating artifact contamination (mainly due to subject motion) rendering the image processing unreliable. T2 images were imputed via the convolutional neural network approach PGAN trained on the UNC-EBDS neonate data (Gilmore et al., 2020) if the corresponding T1 images passed quality control (QC scores of 2–3) and T2 images failed or were of borderline failure in quality control (QC score of 1). Nine T2 scans were imputed based on image quality scoring. Exclusion of participants with imputed T2 data did not alter findings and were therefore included in final analyses (See Supplement 2). The T1w and T2w brain images were corrected for intensity non-uniformity via N4 (Tustison et al., 2010), and rigidly transformed to a pediatric neonate atlas in stereotaxic space (Fonov et al., 2011). Brain masking was performed via the 3D UNet-based infant brain masking tool in ANTSPyNet (Tustison et al., 2021) using both T1w and T2w images jointly, including also extra-axial cerebrospinal fluid spaces in the brain mask. All brain masks were corrected manually in itkSNAP (Yushkevich et al., 2006).

**Fig. 1:**
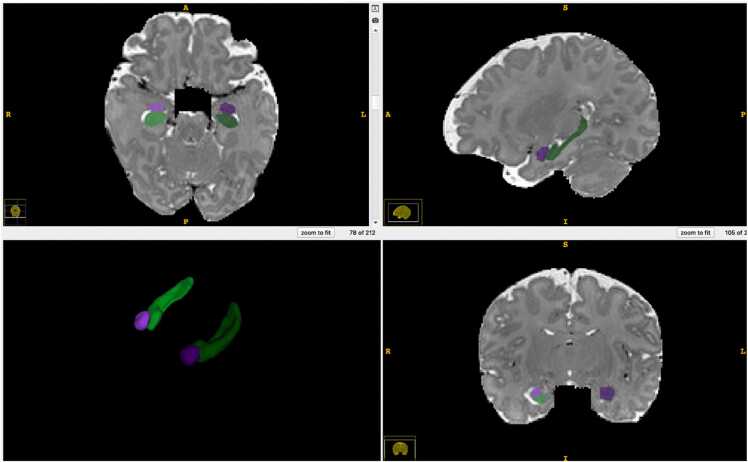
Regions of Interest for the Present Study Note. 3D visualization of representative example of hippocampus and amygdala segmentation on MRI T1 (T2) weighted scan. Purple = amygdala; Green = hippocampus; Left = darker color; Right = brighter color.

Tissue segmentation (into whole brain white matter, gray matter, and cerebrospinal fluid), regional parcellation, as well as hippocampus and amygdala segmentation were performed using a multi-modality (T1w and T2w), multi-atlas segmentation workflow with the in-house, open-source MultiSegPipeline software (Cherel et al., 2015), which employs atlas-registration and label fusion from the ANTs toolset (Tustison et al., 2021). Hippocampus and amygdala regions are defined as in Moog et al., 2018 (see Fig. 1). Total intracranial volume was calculated as the sum of the brain tissue volumes of gray matter (GM), white matter (WM), and cerebrospinal fluid (CSF). Covarying by intracranial volume is common practice (Moog et al., 2018, Demers et al., 2022) in studies comparing regions of interest across individuals as it reduces inter-individual variations in brain volume due to head size. The segmentation quality of all images was visually assessed and rated using a four-point scale (0−3) for anatomical accuracy. No participants were excluded from analyses based on segmentation quality.

#### 2.3.4 Sociodemographic characteristics

Maternal age, socioeconomic status, cohabitation with a partner, marital status, educational attainment, and race and ethnicity were collected via maternal interview at the first prenatal research visit. A family income-to-needs ratio (INR) was calculated by dividing the total reported household income by the poverty threshold corresponding to the number of persons living in the household at the time of study entry, specified by the U.S. Census Bureau for that year.

#### 2.3.5 Pregnancy and birth outcomes

Prenatal obstetric complications, newborn sex, gestational age at birth, and 5-minute Apgar scores were collected from medical records. Additionally, birth weight percentile was calculated using gestational age at birth and newborn sex. Gestational age at birth (GAB) was calculated using early ultrasound measures and/or date of last menstrual period applying the American College of Obstetricians and Gynecologists (ACOG) guidelines. Postconceptional age at scan was computed as GAB plus weeks from birth to MRI scan (Committee on Obstetric Practice, the American Institute of Ultrasound in Medicine, and the Society for Maternal-Fetal Medicine, 2017). Obstetric complications were calculated as a sum score indicating the presence or absence of a series of pregnancy-related complications, including prenatal infection, pregnancy-included hypertension, gestational diabetes, oligohydramnios, polyhydramnios, preterm labor, vaginal bleeding, placenta previa, or anemia (Hobel, 1982). 28% of participants experienced two or more obstetric complications on this index. There were no missing data related to pregnancy and birth outcomes.

### 2.4 Analytical approach

#### 2.4.1 Evaluation of covariates

Bivariate correlations were conducted to test intracranial volume (ICV) and postconceptional age at scan (sum of GAB and weeks from birth to scan), sex at birth, birth weight percentile, prenatal income, parity, and obstetric complications as potential covariates since these have been previously associated with brain volume in the literature (Makropoulos et al., 2016, Moog et al., 2018, Nolvi et al., 2020). Additionally, the relation between hippocampal and amygdala volume and region-specific segmentation quality scores was tested. Variables were included as covariates if they were associated with hippocampal or amygdala volumes at alpha < .05. An independent samples T-test and a one-way ANOVA were additionally conducted to test parity (primiparous, multiparous) and obstetric complications (zero, one, two, or more) during the current pregnancy as potential covariates. Continuous variables were tested in bivariate correlations, see Table 2. Newborn hippocampal and amygdala brain volume did not differ as a function of parity (all *t*s < 1.59, all *p*s > .12), or obstetric complications (all *F*s < 0.65, all *p*s > .52). However, newborn ICV, birth weight percentile, biological sex, and postconceptional age at scan were associated with newborn hippocampal and amygdala brain volume and thus, were included as covariates in all analyses. Sleep quality at any prenatal timepoint was not associated with ICV (all *r*s < .12; all *p*s > .29).

**Table 2:** Bivariate Correlations of Potential Covariates with Amygdala and Hippocampal Volume.

	Right Hippocampal Volume	Left Hippocampal Volume	Right Amygdala Volume	Left Amygdala Volume
ICV	.670***	.550***	.547***	.534***
Infant age at scan	.407***	.398***	.131	.209*
Sex at birth	-.217*	-.126	-.516***	-.351**
BWP	.282**	.167	.136	.300**
GAB	.021	.067	-.031	.130
INR	.107	.047	.081	.095
Region-specific QC score	-.03	.05	.07	-.11

*Note:*

ICV = intracranial volume, BWP = birthweight percentile, INR = income to needs ratio, QC = quality control. Infant age at scan was calculated as the sum of gestational age at birth plus weeks from birth to scan.

* p < .05,

** < .01,

*** p < .001;

#### 2.4.2 Analyses

Multilevel modeling (MLM) was used to test the associations between the trajectories of prenatal maternal sleep quality and hippocampal and amygdala volume using HLM software (Raudenbush et al., 2019). MLM assumes the data collected is nested within persons, allowing for variance to be modeled at multiple, hierarchical levels. At level 1, maternal sleep quality was regressed on linear and quadratic estimates of gestational age. Full information maximum likelihood (FIML) was used for missing data at level 1. FIML is an accurate and unbiased process for addressing missing data within nested and hierarchical models (Black, 2008). At level 2, the variables included were newborn hippocampal and amygdala volumes and established covariates. There was no missing data present at level 2.

We first fit multilevel models to find the best fitting trajectory of maternal sleep quality. Linear and quadratic growth curves were included to test for changes in sleep quality across gestation. Next, we tested whether the trajectory of sleep quality was associated with newborn hippocampal and amygdala volumes employing Bonferroni correction across the two brain regions (p = .025). Biological sex at birth, ICV, postconceptional age, and birth weight percentile were then added to test the relation between prenatal sleep and newborn hippocampal and amygdala volumes in the presence of pertinent birth outcomes and variables. Follow-up analyses were employed to test whether the strength of association varied based on timing of gestation by centering the model early, mid, and late in pregnancy. Sensitivity analyses were performed removing the four individuals with prenatal substance use exposure.

## 3 Results

### 3.1 Trajectories of maternal sleep quality throughout gestation

**Fig. 2:**
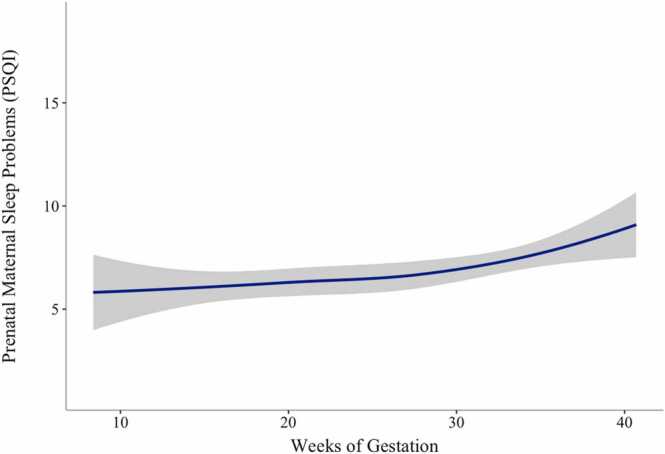
Trajectories of Prenatal Maternal Sleep Quality. Note. Sleep problems measured using Pittsburg Sleep Quality Index (PSQI), higher scores indicate worse sleep quality. Participants contributed up to three timepoints of data. Gestational age calculated using estimated date of delivery and date of PSQI prenatal data collection.

Sleep quality was dynamic over pregnancy with the highest levels of problems in late gestation. Of the linear and quadratic growth curves analyzed, deviance scores indicated that a quadratic growth curve yielded better fit for the trajectories of prenatal maternal sleep quality (Δχ2(1) = 1309.58 – 1300.04 = 9.54, p < .01). The model included fixed and random effects for the intercept and the linear slope, as well as fixed effects for the quadratic curve. Initial sleep quality scores varied across pregnant participants (σ^2^_b0_ = 12.49, *SD* = 3.53, *p* < .001). Similarly, sleep quality trajectories varied across pregnant participants (σ^2^_b0_ = 0.01, *SD* = 0.11, *p* < .001). Results indicate that sleep problems are similar in early and mid-pregnancy with a worsening of sleep quality late in pregnancy. See Table 3 and Fig. 2 for more details.

**Table 3:** Multilevel Growth Models of Prenatal Maternal Sleep Quality.

	Linear Slope	Quadratic Growth
**Fixed Effects**	*b*	*b*
Intercept (*β*_*00*_)	5.61	6.87
Linear Slope (*β*_*10*_)	0.07 * **	-0.11
Quadratic Growth (*β*_*20*_)	–	0.0053 * *
**Random Effects**		
Error (r_e_)	2.63	2.39
Intercept (r_0_)	11.51 * **	12.80 * **
Slope (r_1_)	0.01 * **	0.01 * **

*Note:* *p < .05, * *< .01, * **p < .001. Intercept centered at eight gestational weeks.

### 3.2 Prenatal sleep quality and newborn hippocampal volume

**Fig. 3:**
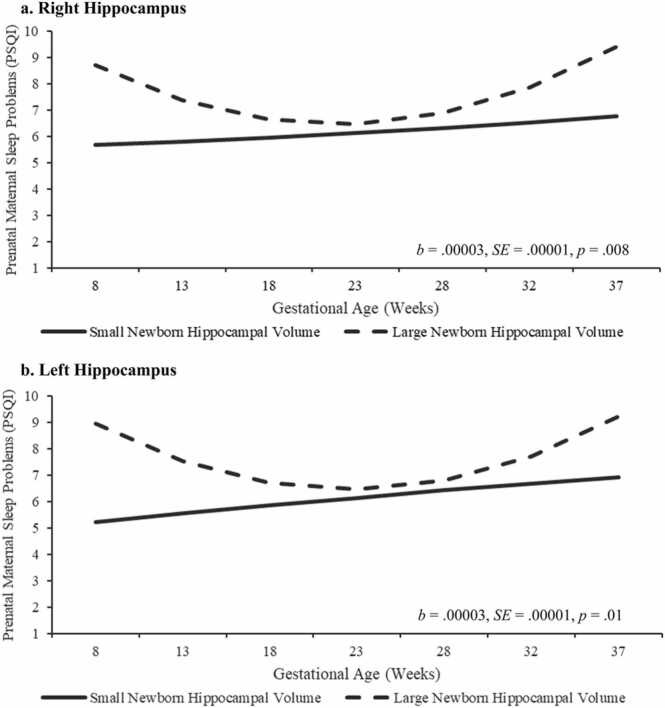
Trajectories of Prenatal Maternal Sleep Quality by Newborn Hippocampal Volume. *Note.* Data were analyzed continuously. Trajectories of prenatal maternal sleep quality are presented by small (−1 SD) and large (+1 SD) newborn hippocampal volume for visualization purposes. Trajectories of prenatal maternal sleep quality predict hippocampal volume, and poorer sleep quality early in pregnancy predicts larger bilateral newborn hippocampal volume, after controlling for ICV, postconceptional age at scan, and sex.

The overall trajectory of sleep quality over gestation was associated with hippocampal volume after inclusion of covariates bilaterally (left: *b* = 0.00003, *p* = 0.013; right: *b* = 0.00003, *p* = .008; See Table 4 and Fig. 3). Follow up analyses to determine whether associations were stronger at certain times in gestation revealed that poorer sleep quality earlier in gestation, but not at mid or later gestation, was associated with larger hippocampal volume. Specifically, lower sleep quality during the early in pregnancy was associated with larger newborn hippocampal volume, bilaterally (left: *b* = 0.01, *p* = 0.018; right: *b* = 0.01, *p* = .025; See Table 4 and Fig. 3). However, sleep later in gestation, was not associated with hippocampal volume (e.g., late gestation left: *b* = 0.002, *p* = 0.24; right: *b* = 0.004, *p* = .11; See Table 6). Additional sensitivity analyses showed that removing the four participants with substance use during gestation did not impact the pattern or significance of findings (See Supplement 1).

**Table 4:** Multilevel Models of Maternal Sleep Quality Across Gestation & Hippocampal Volume.

Model 1 – Right Hippocampus			
		*HippR*	*HippR & Covariates*
**Fixed Effects**	Intercept Centered at 8 Gestational Weeks’ (b_0_)	7.01	7.02
	HippR (b_01_)	0.01 *	0.01 *
	ICV (b_02_)	–	-0.00001
	Postconceptional Age (b_03_)	–	0.28 *
	BWP (b_04_)	–	-0.0001
	Sex (b_05_)	–	-0.45
	Linear Slope (b_1_)	-0.13⸸	-0.12⸸
	HippR (b_11_)	-0.001 *	-0.001 *
	Quadratic Growth (b_2_)	0.006 * *	0.006 * *
	HippR (b_21_)	0.00003 * *	0.00003 * *
**Random Effects**^**a**^	Error (σ^2^_e_)	2.21	2.21
	Intercept (σ^2^_b0_)	13.68 * **	13.22 * **
	Slope (σ^2^_b1_)	0.01 * **	0.01 * **
**Model 2 – Left Hippocampus**			
		*HippL*	*HippL & Covariates*
**Fixed Effects**	Intercept Centered at 8 Gestational Weeks’ (b_0_)	6.97	6.99
	HippL (b_01_)	0.011 *	0.01 *
	ICV(b_02_)	–	-0.00001
	Postconceptional Age (b_03_)	–	0.25
	BWP (b_04_)	–	0.003
	Sex (b_05_)	–	-0.49
	Linear Slope (b_1_)	-0.12⸸	-0.13⸸
	HippL (b_11_)	-0.001 *	-0.001 *
	Quadratic Growth (b_2_)	0.005 * *	0.005 * *
	HippL (b_21_)	0.00003 *	0.00003 *
**Random Effects**^**a**^	Error (σ^2^e)	2.23	2.24
	Intercept (σ^2^_b0_)	13.09 * **	12.84 * **
	Slope (σ^2^_b1_)	0.01 * **	0.01 * **

*Note:* ⸸ *p* < .08, *p < .05, * *< .01, * **p < .001. HippR = Right hippocampus, HippL= Left hippocampus, ICV = Intracranial volume, BWP = Birth weight percentile. ^a^ Intercept and linear slope were tested as random parameters, whereas quadratic growth was tested as a fixed parameter.

**Table 5:** Multilevel Models of Maternal Sleep Quality Across Gestation & Amygdala Volume.

Model 1 – Right Amygdala			
		*AmyR*	*AmyR & Covariates*
**Fixed Effects**	Intercept Centered at 8 Gestational Weeks’ (b_0_)	6.81	6.86
	AmyR (b_01_)	-0.01	-0.02
	ICV (b_02_)	–	-0.000002
	Postconceptional Age (b_03_)	–	0.27⸸
	BWP (b_04_)	–	0.003
	Sex (b_05_)	–	-0.57
	Linear Slope (b_1_)	-0.11	-0.11
	AmyR (b_11_)	0.001	0.001
	Quadratic Growth (b_2_)	0.005 * *	0.005 * *
	AmyR (b_21_)	-0.00003	-0.00003
**Random Effects**^**a**^	Error (σ^2^_e_)	2.41	2.42
	Intercept (σ^2^_b0_)	12.49 * **	12.37 * **
	Slope (σ^2^_b1_)	0.01 * **	0.01 * **
**Model 2 – Left Amygdala**			
		*AmyL*	*AmyL & Covariates*
**Fixed Effects**	Intercept Centered at 8 Gestational Weeks’ (b_0_)	6.79	6.83
	AmyL (b_01_)	-0.01	-0.02
	ICV(b_02_)	–	-0.000002
	Postconceptional Age (b_03_)	–	0.29 *
	BWP (b_04_)	–	0.005
	Sex (b_05_)	–	-0.58
	Linear Slope (b_1_)	-0.10	-0.10
	AmyL (b_11_)	-0.0005	-0.0005
	Quadratic Growth (b_2_)	0.005 * *	0.005 * *
	AmyL (b_21_)	0.00001	0.00001
**Random Effects**^**a**^	Error (σ^2^e)	2.39	2.40
	Intercept (σ^2^_b0_)	12.52 * **	12.29 * **
	Slope (σ^2^_b1_)	0.01 * **	0.01 * **

*Note:* ⸸ *p* < .08, *p < .05, * *< .01, * **p < .001. AmyR = Right amygdala, AmyL= Left amygdala, ICV = Intracranial volume, BWP = Birth weight percentile. ^a^ Intercept and linear slope were tested as random parameters, whereas quadratic growth was tested as a fixed parameter.

**Table 6:** Prenatal Maternal Sleep Quality and Newborn Hippocampal Volume Mid and Late in Pregnancy.

*6a. Prenatal Sleep Quality and Newborn Right Hippocampal Volume Mid and Late in Pregnancy*		
	Mid Pregnancy (20 weeks GW)	Late Pregnancy (30 weeks GW)
**Fixed Effects**	*b*	*b*
Intercept (*β*_*00*_)	6.33	7.04
HippR (*β*_*01*_)	0.002	0.004
Linear Slope (*β*_*10*_)	0.03	0.12***
HippR (*β*_*11*_)	-0.00005	0.0005**
Quadratic Growth (*β*_*20*_)	0.006**	0.006**
HippR (*β*_*21*_)	0.00003**	0.00003**
**Random Effects**		
Error (r_e_)	2.21	2.21
Intercept (r_0_)	9.81***	10.20***
Slope (r_1_)	0.01***	0.01***
6b. *Prenatal Sleep Quality and Newborn Left Hippocampal Volume Mid and Late in Pregnancy*		
	Mid Pregnancy (20 weeks GW)	Late Pregnancy (30 weeks GW)
**Fixed Effects**	*b*	*b*
Intercept (*β*_*00*_)	6.32	7.04
HippL (*β*_*01*_)	0.002	0.003
Linear Slope (*β*_*10*_)	0.03	0.13***
HippL (*β*_*11*_)	-0.0002	0.0004*
Quadratic Growth (*β*_*20*_)	0.006**	0.006**
HippL (*β*_*21*_)	0.00003*	0.00003*
**Random Effects**		
Error (r_e_)	2.23	2.23
Intercept (r_0_)	9.65***	10.39***
Slope (r_1_)	0.01***	0.01***

Note:

* p < .05,

** < .01,

*** p < .001. GW = Gestational weeks; HippR = Right hippocampus; HippL = Left hippocampus. Intercept centered at 20 and 30 gestational weeks for mid and late in pregnancy respectively.

### 3.3 Prenatal sleep quality and newborn amygdala volume

Neither the overall trajectory of prenatal sleep quality (left: *b* = 0.00001, *p* = 0.85; right: *b* = −0.00003, *p* = .59) nor level of sleep quality at any time during pregnancy predicted newborn amygdala volume (left: *b* = −0.02, *p* = .28; right: *b* = −0.02, *p* = .31, See Table 5).

## 4 Discussion

The present study provides new insight into prenatal maternal sleep as a plausible process that contributes to fetal brain development. There is extensive evidence that prenatal experiences shape the maturation of the fetal brain (see Demers et al., 2021a for review). Disruptions in sleep is common occurrence in pregnancy, yet prenatal maternal sleep health has rarely been considered in empirical research as a biological process that may sculpt the fetal brain (Johnson and Louis, 2022, Mindell et al., 2015). The present study provides evidence that the trajectory of maternal sleep quality across gestation is associated with newborn bilateral hippocampal volume and further, that timing of exposure is critical. Timing effects additionally were observed such that poorer sleep quality during the first trimester most strongly predicted larger newborn hippocampal volume, relative to later in gestation. Notably, associations persisted after covarying intracranial volume, age at scan, birth weight percentile, and biological sex at birth. Despite evidence that poor sleep quality is a pervasive public health problem, only a few prior studies evaluate the impact on postnatal function in humans (Ciciolla et al., 2022, Lavonius et al., 2020, Trauman et al., 2015). The present findings provide novel evidence suggesting that sleep disruptions early in gestation may have intergenerational consequences.

Our findings build on previous preclinical studies illustrating that the hippocampus is susceptible to alterations in prenatal maternal sleep. In rodents, experimentally induced prenatal sleep deprivation causes decreases in offspring hippocampal neurogenesis and alterations in hippocampal synaptic plasticity (Peng et al., 2016, Yu et al., 2018, Zhao et al., 2015). We find that hippocampal volume was larger among neonates with fetal exposure to greater prenatal maternal sleep problems. It is not clear why sleep problems are associated with larger neonatal hippocampal volume. The hippocampus is a stress sensitive region, and it is plausible that disrupted sleep modifies the prenatal environment in ways that alter neurogenesis in the hippocampus (Zhao et al., 2015). Consistent with this possibility, recent rodent research indicates links between prenatal sleep disruption and increases in cortical synaptic density (Vanderplow et al., 2022). It is noteworthy that most preclinical studies employ sleep deprivation, a significant stressor different from sleep problems captured within the current study which include difficulty falling asleep, frequent night awakenings, daytime dysfunction, and subjective perceptions of overall sleep quality.

The pathways contributing to changes in hippocampal volume following prenatal maternal sleep disruptions are unknown. Prenatal sleep has been linked to dysregulation in both the Hypothalamic Pituitary Adrenocortical (HPA) axis and immune systems during the prenatal period (Bublitz et al., 2018, Chang et al., 2010), including elevations in circulating cortisol and cytokines (Bleker et al., 2017, Okun et al., 2007). As both the stress and immune systems directly impact fetal neurodevelopment (Curran et al., 2017, Graham et al., 2018, Sandman et al., 2018), disruptions to immune and HPA systems are potential mechanisms contributing to the association between prenatal maternal sleep health and newborn brain structure.

The current study’s longitudinal evaluation of sleep throughout gestation enables us to study the effects of timing of exposure on the fetal brain. Our findings suggest that sleep quality in the first trimester may have the most potent implications for the development of the hippocampus. The hippocampus begins to form as early as the 4th gestational week (Noorlander et al., 2006, Thomason, 2020). This finding that early gestation is a sensitive window for sleep disturbances is consistent with evidence that the developing fetus is particularly susceptible to early gestation maternal stress and sleep perturbations (Davis and Sandman, 2010, Okun et al., 2011). Future work is needed to probe timing effects, as it is plausible that early gestational sleep disruptions exert a greater impact on the developing brain.

We did not observe a link between prenatal sleep quality and newborn amygdala volume. Previous findings have highlighted the susceptibility of the amygdala to prenatal perturbations (Rifkin-Graboi et al., 2013, Prather et al., 2013; Buss, 2012; Demers et al., 2022). Although the link between prenatal maternal sleep and amygdala development is unknown, our findings suggest that prenatal sleep health may not be associated with newborn amygdala volume, it is plausible that as the amygdala continues to develop in the postnatal period, links with prenatal sleep will emerge. Consistent with this possibility, prior work has shown that associations with maternal childhood maltreatment and offspring amygdala volume emerge later in infancy (Khoury et al., 2021).

The present study possessed several strengths and limitations. A crucial strength of this study was the longitudinal and prospective assessment of maternal sleep quality across gestation. Sleep quality changes across pregnancy (Nevarez-Brewster et al., 2022) and differentially predicts newborn brain structure. However, prenatal sleep health was assessed subjectively. Longitudinal and prospective assessment of objective prenatal maternal sleep health using actigraphy or polysomnography would complement subjective sleep perceptions. Additionally, preconception sleep parameters were not assessed and thus, future research could test links between sleep quality from preconception through pregnancy and fetal brain structure. Another strength of this study was the collection of newborn imaging data. We elected to assess the newborn shortly after birth to identify associations with the prenatal environment at a time when postnatal influences have a minimal effect. Only volumetric data was assessed in this study. Future studies could investigate structural and functional connectivity as a function of prenatal maternal sleep health. Offspring neuroimaging was also assessed once. As rapid neuronal growth is particularly salient in the first years of life, future studies should incorporate repeated assessments of offspring brain structure to determine whether the links between prenatal sleep and hippocampal volume persist.

There are several additional future directions to be considered based on the findings of this study. First, behavioral phenotypes that may correlate with the observed alterations in newborn hippocampal volume remain unknown. There is some evidence that larger hippocampal volume and disrupted sleep are related in populations at risk for developmental disorders (MacDuffie et al., 2020). Another recent study found larger hippocampal volume at one-month postpartum increased susceptibility to the benefit of maternal sensitivity at six months on cognitive abilities at two years postpartum (Nolvi et al., 2020). This intriguing finding indicates that a plausible consequence of larger hippocampal volume may be heightened susceptibility to the postnatal environment. Second, sleep disruption is comorbid with maternal physical and mental health including depression (Skouteris et al., 2009), stress (Bublitz et al., 2018), diet (Van Lee et al., 2017), and physical activity (Tan et al., 2020) all of which influence the fetal brain (Dufford et al., 2021). Future work could consider the cumulative and synergistic influence of such processes on the developing brain. Additionally, while the present study established the importance of prenatal sleep health trajectories there is a need for research evaluating sensitive windows during gestation when the fetal brain may be most vulnerable to maternal sleep disruptions as well as patterns of sleep over pregnancy that are most impactful on the fetal brain.

The present findings posit prenatal maternal sleep health as a process implicated in the programming of the developing fetal brain. This work lays foundational knowledge for future studies to further understand the intergenerational impact of prenatal sleep health. Recent findings suggest cognitive behavioral therapy for insomnia can improve subjective and objective sleep during pregnancy while also improving prenatal maternal mood (Tomfohr-Madsen et al., 2016). Ultimately, the prenatal period is an optimal time in development for intervention and prenatal maternal sleep is amenable to intervention (Davis and Narayan, 2020a). Thus, an improved knowledge of the intergenerational impact of prenatal sleep disruptions may support the development of prenatal sleep interventions.

## Funding

This work is supported by the 10.13039/100000002National Institutes of Health: R01MH109662 [EPD, BLH] R01HL155744 [EPD, BLH], R01MH111944 and R01HD053000 (JHG), P50. HD103573, U54 HD079124 (MAS), T32 Training fellowships MH125572 [CHD], MH106440 [MMB], and diversity supplement 3R01HL155744-01S1 [EPD, Recipient: MNB].

## Declaration of Competing Interest

The authors declare that they have no known competing financial interests or personal relationships that could have appeared to influence the work reported in this paper.

## Data Availability

Data is available upon request.

## Footnotes

2 Participants self-identified categorizes of race and ethnicity at time of enrollment. We recognize the ways in which such imposed categorization can minimize the complexities of human experience, and advertently or inadvertently, cause harm. These categorizations should be used with caution within academic realms and while interacting with participants and the public.
