# Supplementary material for: Longitudinal and prospective assessment of prenatal maternal sleep quality and associations with newborn hippocampal and amygdala volume
Source: Dev Cogn Neurosci. 2022 Nov 9;58:101174. doi: 10.1016/j.dcn.2022.101174 (PMC9661438; doi:10.1016/j.dcn.2022.101174)
Supplement: Supplementary file 1 — Supplementary material. [file mmc1.docx]

**Supplement 1**

*Prenatal Maternal Sleep Quality & Hippocampal Volume Excluding Prenatal Substance Users (N = 90)*

|  |  | *HippR* | *HippL* |
| --- | --- | --- | --- |
| **Fixed Effects** | Intercept Centered at 8 Gestational Weeks’ (b_0_) | 7.15 | 7.14 |
|  | Hippocampus (b_01_) | 0.008* | 0.01* |
|  | Linear Slope (b_1_) | -0.13* | -0.13* |
|  | Hippocampus (b_11_) | -0.001** | -0.001** |
|  | Quadratic Growth (b_2_) | 0.006** | 0.006** |
|  | Hippocampus (b_21_) | 0.00003** | 0.00003** |
| **Random Effects^a^** | Error (σ^2^_e_) | 2.21 | 2.21 |
|  | Intercept (σ^2^_b0_) | 14.30*** | 13.81*** |
|  | Slope (σ^2^_b1_) | 0.01*** | 0.01*** |

*Note:* *p<.05, **<.01, ***p<.001. HippR = Right hippocampus, HippL= Left hippocampus. ^a^ Intercept and linear slope were tested as random parameters, whereas quadratic growth was tested as a fixed parameter.

**Supplement 2**

*Prenatal Maternal Sleep Quality & Hippocampal Volume Excluding T2 Imputed Data (N = 85)*

|  |  | *HippR* | *HippL* |
| --- | --- | --- | --- |
| **Fixed Effects** | Intercept Centered at 8 Gestational Weeks’ (b_0_) | 7.04 | 6.98 |
|  | Hippocampus (b_01_) | 0.01* | 0.01* |
|  | Linear Slope (b_1_) | -0.13⸸ | -0.12⸸ |
|  | Hippocampus (b_11_) | -0.001* | -0.001* |
|  | Quadratic Growth (b_2_) | 0.005** | 0.006** |
|  | Hippocampus (b_21_) | 0.00003** | 0.00003* |
| **Random Effects^a^** | Error (σ^2^_e_) | 2.11 | 2.14 |
|  | Intercept (σ^2^_b0_) | 15.08*** | 14.69*** |
|  | Slope (σ^2^_b1_) | 0.01*** | 0.01*** |

*Note:* ⸸ *p* < .08, *p<.05, **<.01, ***p<.001. HippR = Right hippocampus, HippL= Left hippocampus. ^a^ Intercept and linear slope were tested as random parameters, whereas quadratic growth was tested as a fixed parameter.

**Supplement 3**

*Trajectories of Prenatal Maternal Sleep Quality Including All Data Points*


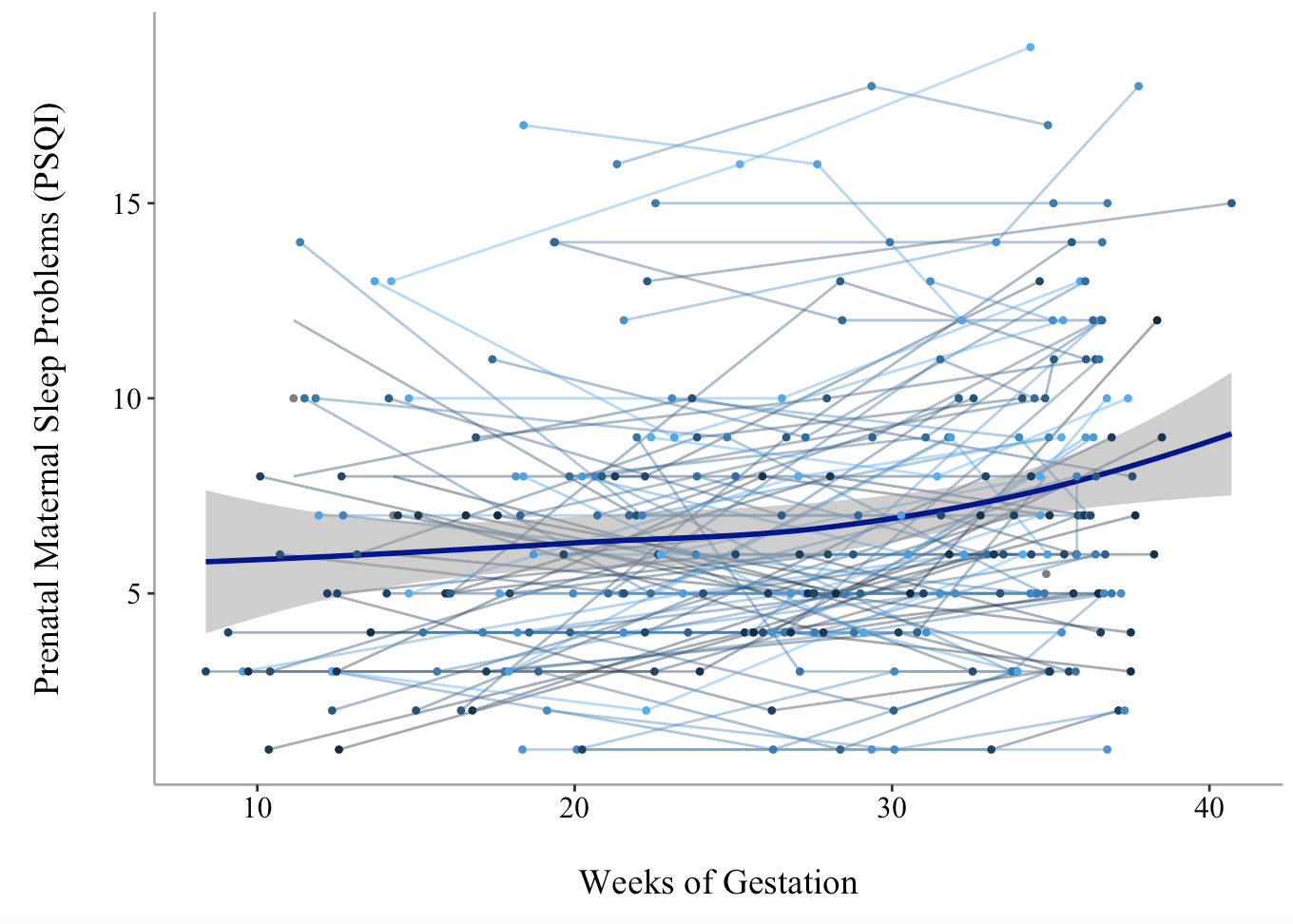


*Note.* Sleep problems measured using Pittsburg Sleep Quality Index (PSQI), higher scores indicate worse sleep quality. Participants contributed up to three timepoints of data.
